# Multisensory contribution in visuospatial orientation: an interaction between neck and trunk proprioception

**DOI:** 10.1007/s00221-021-06146-0

**Published:** 2021-06-13

**Authors:** Jason McCarthy, Patricia Castro, Rachael Cottier, Joseph Buttell, Qadeer Arshad, Amir Kheradmand, Diego Kaski

**Affiliations:** 1 Regional Neurological Rehabilitation Unit, Homerton University Hospital, London, UK; 2 Neuro-otology Unit, Department of Brain Sciences, Imperial College London, London, UK; 3 inAmind Laboratory, Department of Neuroscience, Psychology and Behaviour, University of Leicester, Leicester, UK; 4 Department of Neurology, The Johns Hopkins University School of Medicine, Baltimore, USA; 5 Department of Clinical and Movement Neurosciences, Centre for Vestibular and Behavioural Neuroscience, University College London, London, UK; 6 Facultad de Medicina Clínica Alemana, Universidad del Desarrollo, Santiago, Chile

**Keywords:** Subjective visual vertical, Visual dependence, Cervical, Proprioception, Body, Graviception

## Abstract

A coherent perception of spatial orientation is key in maintaining postural control. To achieve this the brain must access sensory inputs encoding both the body and the head position and integrate them with incoming visual information. Here we isolated the contribution of proprioception to verticality perception and further investigated whether changing the body position without moving the head can modulate visual dependence—the extent to which an individual relies on visual cues for spatial orientation. Spatial orientation was measured in ten healthy individuals [6 female; 25–47 years (SD 7.8 years)] using a virtual reality based subjective visual vertical (SVV) task. Individuals aligned an arrow to their perceived gravitational vertical, initially against a static black background (10 trials), and then in other conditions with clockwise and counterclockwise background rotations (each 10 trials). In all conditions, subjects were seated first in the upright position, then with trunk tilted 20° to the right, followed by 20° to the left while the head was always aligned vertically. The SVV error was modulated by the trunk position, and it was greater when the trunk was tilted to the left compared to right or upright trunk positions (*p* < 0.001). Likewise, background rotation had an effect on SVV errors as these were greater with counterclockwise visual rotation compared to static background and clockwise roll motion (*p* < 0.001). Our results show that the interaction between neck and trunk proprioception can modulate how visual inputs affect spatial orientation.

## Introduction

Appropriate postural control requires an accurate internal representation of spatial orientation assimilated from multiple sensory sources (Massion 1994; Horak 2006). This can be quantified using a subjective visual vertical (SVV) task in which the direction of gravity is used as a reference to report perceived spatial orientation (Dichgans et al. 1972; Gresty et al. 1992; Brandt et al. 1994; Bronstein et al. 2003; Barra et al. 2010; Kheradmand and Winnick 2017). It has been established that such graviceptive information arising from the vestibular organs integrates with visual inputs and body proprioception, with the net result contributing to the multisensory integration required for coherent spatial orientation (Mittelstaedt 1996; Karnath et al. 2000; Barra et al. 2010).

Perception of spatial orientation is primarily processed in a head-in-space reference frame, but there are also significant effects from changes in trunk and neck positions (Wade 1968; Wade and Day 1968; Mittelstaedt 1996; Karnath et al. 2000; Guerraz et al. 2003; Barra et al. 2010; Clemens et al. 2011; Otero-Millan and Kheradmand 2016). Such diverse sensory contribution is significant as it allows the brain to modulate spatial perception in accordance with the current state of body position for optimal interaction with the environment. Importantly, verticality perception is itself influenced by our changing visual environment (Dichgans et al. 1972). The degree of visual dependence—the extent to which an individual relies on visual inputs—is itself dependent upon the reliability of the sensory signals involved in spatial orientation and postural control (Bronstein 1995; Tjernström et al. 2019). For example, in microgravity where inertial vestibulo–proprioceptive cues are reduced, the weight given to vision for perceived body orientation is potentiated (Cheung et al. 1990). Likewise, changes in the body position under normal circumstances must also influence the weight of visual inputs in spatial orientation. Here, we examined the contribution of proprioceptive information in this process and asked whether changes in the trunk positions alone (i.e. without changing the head position) can modulate visual dependence. To address this aim, we disassociated body position from any graviceptive information arising from the vestibular system while measuring individuals’ ability to visually evaluate upright vertical.

## Material and methods

### Participants

This study was approved by the Wales Ethics Research Committee (Ref: 17/WA/0097). 10 healthy individuals (six female) gave informed consent to participate in the study. All subjects underwent a clinical interview and neurological examination to ensure there were no neurological, ontological, ophthalmological, or musculoskeletal abnormalities Ages ranged from 25 to 47 (mean 32.8; SD 7.8 years). All participants were right-handed (self-reported). Exclusion criteria included significant visual impairment (not correctable with glasses), neurological impairment (including history of seizure, stroke, frequent migraines, or vestibular impairment), and any current or historical neck pain.

### SVV equipment

Two identical mobile phones (Samsung S7, Samsung Electronics Group, South Korea) using the Android operating system and communicating via Bluetooth protocol were required to run the visual vertical assessment software. The first phone ran the VR component of the software and acted as a display, residing within a VR headset (Samsung Oculus Gear, Samsung Electronics Group, South Korea) worn by the participant. The second phone ran the control component of the software and was used to setup and monitor the VR environment, and record the result (Ulozienė et al. 2017, 2020). The device was calibrated prior to starting data acquisition for each participant to ensure measurement of true earth vertical. Participants used a wireless gamepad joystick controller (DragonSlay TripleMode Gamepad, DragonSlay, UK) to interact with the VR environment and align the arrow to perceived earth vertical.

### SVV trials

Participants wore the VR headset to view a red arrow on a plain black background (static). At the start of each test the VR software randomly orientated the arrow to a start position of 10°–15° (0.1° increments) left or right of the gravitational vertical. Using the gamepad controller participants were given the following instructions: “On the screen you will see a red arrow on a blank background. Using the controller point the arrow as close to a vertical position (floor to ceiling). Tell [the researchers] when you are satisfied the arrow is pointing directly upright. You will then be given a further opportunity to ensure you are satisfied with the arrow’s position. [The researchers] will record the position of the arrow before moving onto the next test. There will be ten tests against the blank background before we move on to the next set.” Participants verbally indicated that they were satisfied with the vertical orientation of the arrow and any discrepancy between the end position and 0° was recorded (with an accuracy of 0.1°). SVV errors were automatically calculated and outputted by the software as the difference between adjusted arrow orientation and true vertical. Ten repetitions were carried out. Participants then completed a second set of ten trials in another condition, this time with the arrow set against the same black background but with ten spheres rotating in a clockwise (CW) direction at constant velocity of 30°/s (Ulozienė et al. 2017). Participants were asked to confirm the change of visual context, before continuing the process. After competition of this condition, a third set of ten trials were then completed with the same spheres rotating at the same velocity but in the counterclockwise (CCW) direction. The use of rotating background on SVV has been widely used to study the effect of visual stimulation upon spatial orientation and verticality perception (Dichgans et al. 1974; Cheung et al. 1990; Cousins et al. 2014; Roberts et al. 2016; Bednarczuk et al. 2020; Ulozienė et al. 2020).

### Body tilts

**Fig. 1:**
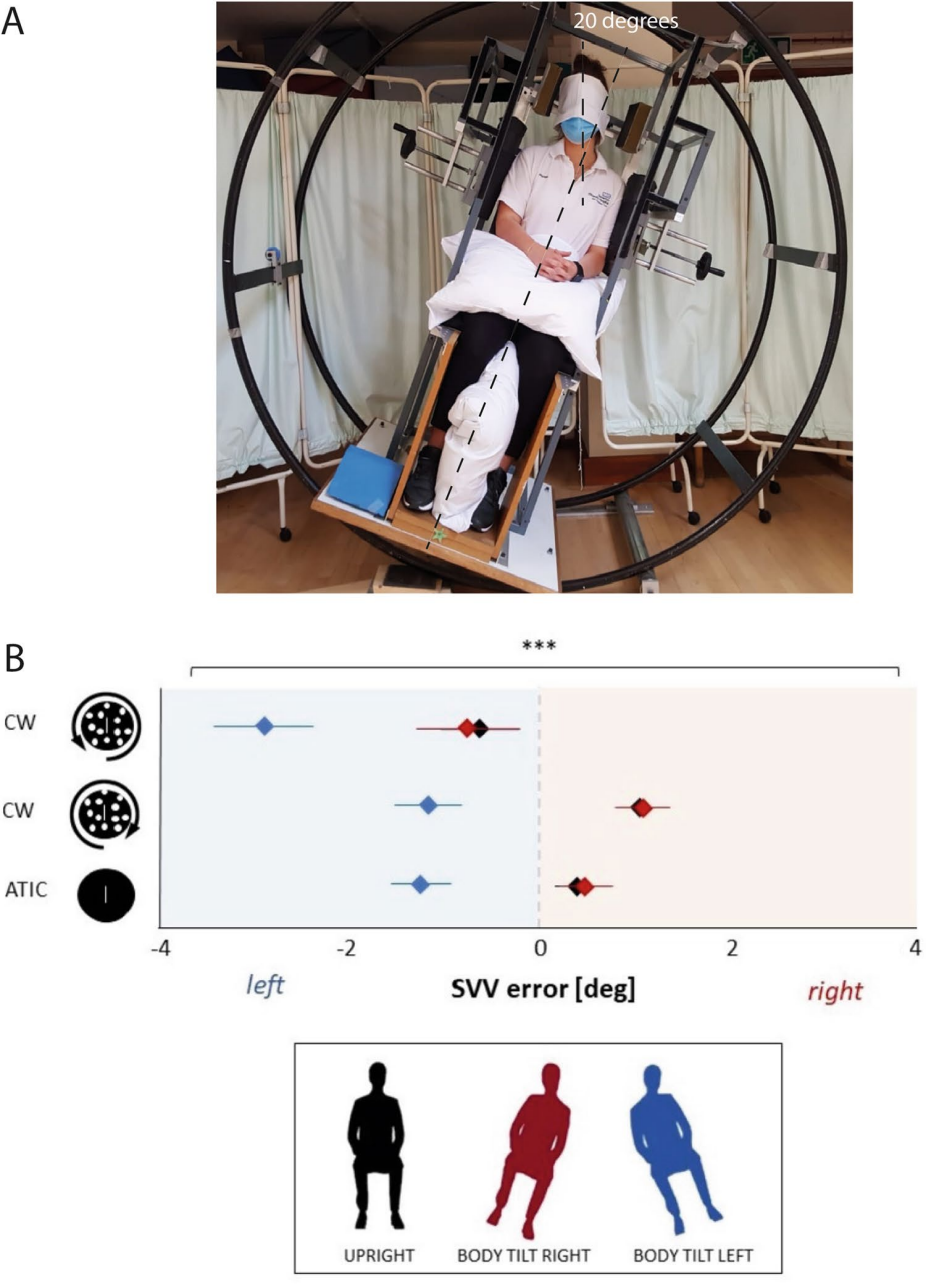
**a** Experimental setting. Subjects were in a seated position on the ‘wheel’ wearing VR goggles (not shown). Here the entire wheel is rotated so that the trunk is tilted to the left by 20°, but the head was maintained in an upright position through passive leftwards neck side-flexion. The head was secured comfortably in this position adjustable padded head clamps attached to the wheel. Cushions were placed around the participant’s pelvis, trunk, and legs to prevent movement of the body and limbs when the wheel was rotated. Subjects completed the SVV task presented through the VR headset. For rightward body tilts the head was maintained in the upright position through leftward neck side-flexion. **b** SVV mean error in degrees for all body tilt conditions (upright in black, right in red and left in blue; patient's viewpoint) and all stimuli rotation direction (static, CW and CCW). The central line in the *X* axis represents 0° error or no SVV deviation, and a value placed to the right or to the left of this bar represents a deviation of the vertical alignment of the rod towards the right or left, respectively. Diamonds represent the mean value and bars represent the standard error

For the first tilt condition, i.e. Upright, participants were seated within a drum-like metal tilting apparatus (‘wheel’) (Perennou et al. 2008) and positioned at midline in upright orientation. Bilateral headrests provided support and stability during testing. Cushions were added to the lower limbs, pelvic area and trunk to ensure the trunk and legs were secured and comfortably restrained (Fig. 1A). In this position, subjects completed the SVV trials as described above. Once these three sets of ten SVV trials were completed in the Upright position, the wheel was manually rotated by the examiner to tilt the participant’s trunk to 20° right or left (i.e., 20R or 20L). Drum rotational tilt was measured with an inertial inclinometer with a resolution of 0.5°. In these body tilt positions participants were assisted to side-flexed the neck 20° to the contralateral side of the trunk tilt to bring their head back to upright (0°). The head was secured comfortably in this position using adjustable padded head clamps attached to the wheel (Fig. 1A). Participants then completed the three sets of ten SVV trials (static, CW and CCW) in the right body tilt, and finally subjects completed the task in the left body tilt positions.

Each set of thirty tests (static, CW, CCW) was completed in approximately 15–20 min. Prior to each reorientation of trunk position, participants were permitted to rest at midline with the VR headset removed (i.e., with their head and trunk aligned back to upright) for 3–5 min before being moved to the next position. This gave an opportunity for visual reorientation to upright vertical. Due to timing restrictions, not all participants were able to complete testing in the three positions during a single session, but the thirty trials carried out in each postural set were always completed in the same sitting.

In addition to headrests, two further methods of ensuring a consistent head position were employed. First, participants were advised the VR red arrow would turn blue if head alignment deviated from either the horizontal or vertical axis and they were asked to alert the examiners if this occurred. Second, a rotational inclinometer (Starrett Exact RS-492-005) measuring the horizontal angle of the VR headset allowed constant monitoring of head position by the examiners. Soft padded materials placed around the participants’ lower limbs helped maintain lower body position and alleviate potential proprioceptive interference from below the pelvis.

### Data analysis

All trials were averaged for each subject, then a single error value per body tilt condition and per visual background rotation direction (CW, CCW) was analysed for each participant. The data complied with the normality and sphericity assumptions. To identify whether body tilt, isolated from head tilt, affects SVV estimates we conducted a repeated measures ANOVA. We compared (1) the effect of trunk tilt and (2) the effect of visual background direction on SVV error as main factors, as well as the interaction between these two. To avoid any statistical bias in the analysis, a multiple comparisons correction was implemented when ANOVAs were used, correction for three comparisons in the one factor ANOVAs and for nine comparisons when two factors were included in the model. When a main effect was observed in the repeated measures ANOVA, individual paired *t* tests with multiple comparison correction were run to identify that driver of this main effect. Statistical analysis was carried out using SPSS statistics 25.0.

## Results

With the trunk upright, the average SVV errors were 0.380° (SD 1.07) for the static background, 1.028° (SD 0.98) for the CW, and − 0.609° (SD 1.84) for the CCW conditions. The average SVV errors with the right trunk tilt were 0.514° (SD 1.37) for the static, 1.11° (SD 1.37) for the CW and − 0.711° (SD 2.64) for the CCW conditions. The average SVV errors with the left trunk tilt were − 1.257° (SD 1.51) for the static, − 1.193° (SD 1.66) for the CW and 2.891° for the CCW conditions (SD 2.55; Fig. 1B). Significant main effects of trunk tilt [*F*_(2, 18)_ = 34.961, *p* < 0.001, *ŋ*^2^ = 0.795] and the direction of background roll motion [*F*_(2, 18)_ = 9.931, *p* < 0.01, *ŋ*^2^ = 0.525] were observed. However, no significant interaction was seen between these two factors [*F*_(4, 36)_ = 1.638, *p* = 0.186, *ŋ*^2^ = 0.154]. A pairwise comparison showed that error was greater when the trunk was tilted to the left, regardless of whether the background was static (*p* < 0.001) or moving in CW (*p* < 0.001) or CCW (*p* < 0.001) directions. Errors observed in the Right tilt condition were not significantly different from those observed without tilt (Upright condition). Similarly, a paired analysis with correction revealed that SVV errors were significantly larger for the CCW compared to CW in all body tilt positions (*p* < 0.05).

A visual dependence value was calculated by subtracting the SVV error during CCW or CW from static SVV trials (Cousins et al. 2014). Visual dependence was significantly skewed to the left in the left trunk tilt condition compared to right tilt and upright for both CW and CCW [*F*_(2, 18)_ = 21.879, *p* < 0.001 *ŋ*^2^ = 0.709 for CW and *F*_(2, 18)_ = 14.860, *p* < 0.001, *ŋ*^2^ = 0.623 for CCW]. Pairwise comparisons with correction revealed that the main effect of condition (body tilt) was driven by left body tilt, such that SVV errors during left body tilt were significantly different from right tilt (*p* < 0.001) and upright (*p* < 0.01) positions, but the right tilt was not significantly different from the upright position (*p* = 0.602). Finally, we averaged the visual dependence values for both rotation directions (CW and CCW) to obtain the overall bias for each body position. A significant effect of tilt was observed, showing again a larger SVV error towards the left in the left trunk tilt (− 1.46°) position compared to the upright (− 0.17°) and right (− 0.31°) trunk tilt positions [*F*_(2, 18)_ = 21.910, *p* < 0.001, *ŋ*^2^ = 0.709].

To investigate sensory interactions mediating the visual dependence we compared the results with congruent direction of visual stimulation and body tilt (e.g., the CW condition and right body tilt position) against incongruent visual stimulation and body tilt (e.g., CW direction and the left body tilt position). There was no significant difference in visual dependence whether the direction of visual rotation (i.e., CW or CCW condition and body tilt i.e., right or left tilt) were congruent or incongruent (− 0.89° for congruent and − 0.95° for incongruent *p* = 0.676).

We compared static SVV estimates for the last trial with the first trial in upright, right, and left body tilt conditions to identify whether any adaptation had taken place. Differences in SVV error between the first and last trial were not significant, suggesting there was no adaptation or learning effect during the task.

## Discussion

Our results show that changes in the trunk position alone (i.e. without changing the head position) can modulate visual-induced SVV errors. Such visual dependence was not affected by whether the direction of visual stimulation and trunk tilt were congruent or not, and overall the error was larger with the trunk tilted to the left than when the trunk was tilted to the right or upright.

### Body proprioception for upright perception

SVV errors reflect the challenge for the brain in maintaining a common reference for spatial orientation based on the incoming sensory information encoding the eye, head and body positions (Tarnutzer et al. 2010). In the upright position, SVV errors typically remain within two degrees of earth vertical, but with lateral head or body tilts there are systematic errors in perceived upright orientation which do not correspond with the perception of body tilt (Van Beuzekom and Van Gisbergen 2000; Kaptein and Van Gisbergen 2004). When the head is tilted, the compensatory change of the eye position driven by the vestibulo–ocular reflex directly affects the orientation of the images on the retina, but any indirect effect of neck or trunk tilt on how visual inputs are integrated into spatial orientation presumably occur through the process of multisensory integration. Our results indeed show that changes in the trunk position can affect this multisensory process and alter visuospatial orientation even when the head remained in an upright position.

When the whole-body is tilted in the roll plane, SVV normally becomes less accurate such that at small tilt angles (< 60°) the error is often in the opposite direction of the whole-body tilt; a phenomenon known as the Müller or E-effect (Aubert 1861; Müller 1916; Witkin and Asch 1948; Kheradmand et al. 2016). Our results, however, show that the trunk tilt alone led to SVV errors in the same direction of the tilt. The errors were larger during the left tilt suggesting a lateralised effect of trunk position (Fig. 1B). In line with this finding asymmetric SVV errors have been also recorded during neck vibration, with maximal errors during left side vibration of the neck when the head was tilted to the right side (McKenna et al. 2004). In another experiment in healthy participants, vibration applied to the neck resulted in significant body rotations in a sequence of stepping-in-place tasks, but only when vibration was applied to the left sided neck muscles (Malmström et al. 2017) (inducing stretching of neck muscle spindles as would occur during right head roll tilt). Our data align with these findings, and further support the notion that an interaction of neck and trunk afferents can modulate verticality perception.

### Visual dependence and body position

In the process of sensory integration for spatial orientation, retinal information is highly accurate in detecting orientation of a visual stimulus while other sensory inputs are inherently noisier (Vandenbussche et al. 1986; Tarnutzer et al. 2009). Thus, visual cues can have a great influence on one’s perception of spatial orientation, and the extent of such reliance can be affected by other sensory information in both health and disease (Bronstein 1995; Cousins et al. 2014; Roberts et al. 2016; Bednarczuk et al. 2020). For example, with changes in the head tilt position, where vestibular inputs become less reliable, visual cues usually have stronger influence on SVV responses (e.g., a static tilt of the visual background or visual motion) (Dichgans et al. 1974; Young et al. 1975; Vingerhoets et al. 2009). In this context, SVV errors were found to be larger if the head was tilted in the opposite direction of visual rotation. Our results show that verticality perception is indeed dependent upon multisensory integration and in this process the position of the trunk can modulate visuospatial orientation. Such trunk effects could be related to an interplay between cervical and trunk positions, with either proprioceptive organs in the musculature of the neck or stretch receptors within the larger blood vessels between head and trunk as potential sources of afferent cervical information. Neck side-flexion has been observed to influence perceptual tasks in supine postures where vestibular input is negated (Guerraz et al. 2003). Mechanoreceptors and baroreceptors monitor stretch within the cervical and upper thoracic vasculature, with the vagus and glossopharyngeal nerves communicating from the aortic arch and the carotid sinus, respectively (Pirahanchi and Bordoni 2019). The specific origin of graviceptive information in the trunk has been speculated upon (Mittelstaedt 1996; Barra et al. 2010) and most likely stems from body proprioception. Following observations of subjects under centrifugal force, Mittelstaedt (1996) proposed two somatosensory sources of graviception: the first system originating from the lower thoracic region of T11 (and which is seen to be abolished in subjects with nephrectomies); and a second system in the region of C6 which responded to increased inertia concomitant with blood flow moving cephalically, leading to speculation of phrenic or vagus nerve involvement.

When the head is tilted laterally, there is a compensatory torsional eye movement in the opposite direction of the head tilt. This vestibulo–ocular response is far less than the actual degree of head tilt and is a distinct source of error in the SVV response (Otero-Millan and Kheradmand 2016). With the trunk tilt alone, however, there is minimal change in the torsional eye position (Ott 1992). Accordingly, we do not expect such a significant effect of trunk tilt on torsional eye position in our results even though we did not directly record the eye position in the present study. Therefore, we postulate that with changes in the trunk tilt position, the SVV responses are primarily modulated by the proprioceptive inputs.

The asymmetric effect of trunk tilt on visual dependence suggests a lateralized effect of neck and trunk proprioceptive interaction and how they can modulate the influence of visual inputs in spatial orientation. Lateralised effects such as these have also been observed in experiments probing visuo–vestibular interactions (Arshad et al. 2013) and imply a degree of hemispheric dominance for spatial orientation (Arshad et al. 2014). Such lateralisation is intriguing and may relate to asymmetric neuronal networks at the cortical level. Despite the strong lateralisation of the effect we report, we unfortunately did not ascertain a laterality index for each participant. Understanding the pathophysiology of sensory contributions to perception of spatial orientation has potential clinical application. In this context, absent or diminished proprioceptive information can lead to postural instability (Lord et al. 1994; Horak 2006), while the disruption in central integration with other sensory modalities may result in aberrant postural control, such as that of Pusher Syndrome, a phenomenon observed in some stroke survivors which manifests as “pushing” towards the hemiplegic side with the unaffected upper and lower limb, leading to instability in sitting and standing (Karnath et al. 2000; Barra et al. 2010). Understanding the visual, vestibular, but also proprioceptive factors that influence verticality perception could help direct therapeutic interventions in such patient populations.

## Limitations and future directions

The head tilt condition alone was not included in the current design as this effect has been widely investigated previously (Wade 1968; Dichgans et al. 1974; Young et al. 1975; Tarnutzer et al. 2010; Kheradmand et al. 2016; Otero-Millan and Kheradmand 2016), and reference to head tilts with upright body in the discussion is thus inferred from previous published data rather than a direct observation in our study.

Our present results suggest an effect of trunk tilt on how visual inputs are integrated into spatial orientation. This novel finding is a direct evidence towards the multisensory aspect of spatial orientation and how changes in one sensory modality can alter processing of the perceptual output. Future research should direct attention upon decomposing this effect to elucidate the contribution of each component, including body and neck proprioceptors, but also musculoskeletal factors that may be differentially affected by gravity.

Further research should also dissociate the motor aspect from reporting the perceptual responses, to identify whether SVV errors differ when perceptual responses are recorded directly.

## Conclusions

In summary, we found a significant contribution of trunk proprioception upon perception of visuospatial orientation and our results suggests a lateralized effect of trunk tilt position in this process. Our data suggests a role for extra-cranial pathways in the perception of verticality that may contribute to the underlying mechanism of neurological disorders of perceptual verticality, with possible implications for rehabilitation.

## Data Availability

The datasets generated during the current study are not publicly available but are available from the corresponding author on reasonable request.
